# Estimating the secondary attack rate and serial interval of influenza-like illnesses using social media

**DOI:** 10.1111/irv.12321

**Published:** 2015-06-09

**Authors:** Elad Yom-Tov, Ingemar Johansson-Cox, Vasileios Lampos, Andrew C Hayward

**Affiliations:** a Microsoft ResearchHerzeliya, Israel; b Department of Computer Science, University College LondonLondon, UK; c Department of Computer Science, University of CopenhagenCopenhagen, Denmark; d Farr Institute of Health Informatics Research, University College LondonLondon, UK

**Keywords:** Influenza-like illness, secondary attack rate, social media

## Abstract

**Objectives:**

Knowledge of the secondary attack rate (SAR) and serial interval (SI) of influenza is important for assessing the severity of seasonal epidemics of the virus. To date, such estimates have required extensive surveys of target populations. Here, we propose a method for estimating the intrafamily SAR and SI from postings on the Twitter social network. This estimate is derived from a large number of people reporting ILI symptoms in them and\or their immediate family members.

**Design:**

We analyze data from the 2012–2013 and the 2013–2014 influenza seasons in England and find that increases in the estimated SAR precede increases in ILI rates reported by physicians.

**Results:**

We hypothesize that observed variations in the peak value of SAR are related to the appearance of specific strains of the virus and demonstrate this by comparing the changes in SAR values over time in relation to known virology. In addition, we estimate SI (the average time between cases) as 2·41 days for 2012 and 2·48 days for 2013.

**Conclusions:**

The proposed method can assist health authorities by providing near-real-time estimation of SAR and SI, and especially in alerting to sudden increases thereof.

## Introduction

Understanding the transmission dynamics of influenza and influenza-like illnesses (ILI) is vital for deciding on public health strategies to reduce the impact of the virus. An important parameter in the spread of pandemic diseases is their secondary attack rate (SAR): the probability that infection occurs among susceptible persons within a reasonable incubation period following known contact with an infectious person or an infectious source.^1^ A further important parameter for influenza transmission models widely used to design control measures is the serial interval (SI): the time between symptom onset of a primary case and symptom onset of its secondary cases.

Collecting the necessary data for computing SAR and SI entails the tracking of relatively large populations or identification of cases and follow-up of their contacts and is compounded by the fact that the majority of people suffering from influenza do not seek medical attention. For example, only 17% of laboratory-confirmed cases in a large community cohort in England sought medical attention.^2^ Thus, periodic surveys are sometimes employed for data collection,^2^ although these require a large effort by researchers or health authorities and the public completing them.

Public health bodies monitor influenza based on those who seek medical attention, but this surveillance provides no direct information on transmissibility. Some countries also plan more detailed ascertainment of cases and their contacts during pandemics but even these studies may have difficulty in estimating secondary attack rates within households because identified cases and their contacts are likely to receive antivirals.^3^ Selection bias is inherent in outbreak investigations which may also overestimate transmissibility.^4^ A mechanism to routinely monitor an indicator of influenza transmissibility, such as the SAR, and of SI using standardized methodology that could be used on an international scale would therefore be an important tool to guide pandemic response.^5^

Behavioral data from the Internet in general, and social media in particular, are known to correlate well with various health behaviors. The severity of influenza was tracked using search engines,^6^ advertisements^7^ and social media.^8^ Although the accuracy of the first of these has been criticized,^9^ partially for its sensitivity to media attention to seasonal flu, it remains an inexpensive and near-real-time tool for monitoring influenza load across multiple geographies.

Tracking influenza load through Internet activities provides a more sensitive sensor than that afforded by hospitalizations and doctor visits because it serves as a window into people's health concerns even when these do not warrant a visit to medical facilities. They are also advantageous over surveys because they can be collected with a much smaller effort. The drawbacks of these data are that they cannot be directly verified (e.g., using genetic testing for the specific strain of the virus from which a person is suffering), use ambiguous language, and that people sometimes overdiagnose themselves.^10^ Here, we study a specific type of SAR known as the familial or household secondary attack rate (fSAR). fSAR is defined as the probability that at least one household contact becomes a secondary case given that one of the family members was infected.^5^ We estimate fSAR by observing reports of influenza-like illness and its symptoms in social media, and whether they pertain to the reporting user themselves or to their immediate family members.

## Methods

### Data

#### Twitter data

We collected all messages from the Twitter social network, also known as tweets, originating from England during two periods: October 1, 2012, to April 30, 2013, and October 1, 2013, to April 30, 2014. We refer to these datasets as the 2012 and 2013 flu seasons, respectively. Tweets were identified as originating in England if they had GPS coordinates embedded in them, and these coordinates were within England. A total of 80 950 393 tweets from 883 342 users were found in tweets posted during the 2012 season, and 133 569 081 tweets from 1 230 678 users were found for the 2013 season.

#### Survey data

We conducted a survey among 93 self-reported Twitter users, recruited through the CrowdFlower crowdsourcing platform. Self-reported age of 94% of the participants was between 18 and 44 years, and 83% were males. These data were used to validate our hypotheses, as detailed in the Results.

### Identifying illness tweets

Twitter content was filtered to identify tweets related to ILI in a two-step process. First, we found tweets which contained highly indicative terms. Then, we applied a predictor to assess the probability that these tweets mentioned that a specific person (either the account owner or a family member) was suffering from ILI. In the following, we describe both stages.

To identify Twitter messages that were likely related to ILI, we constructed a large set of ILI-related terms and then narrowed them to contain only the most informative of these terms. We began by manually crafting a list of 36 textual markers (or *n*-grams) related to or expressing symptoms of ILI by browsing through related Web pages (on Wikipedia and health-oriented Web sites). Then, using those markers as a seed, we extracted a set of frequent *n*-grams (with *n *≤* *4) that co-occurred with them in a Twitter corpus containing approximately 30 million Tweets published in February and March 2014 and geo-located in the UK. Consequently, the list of markers was expanded to a set of *M *=* *217 *n-*grams.

Using the large set of terms, we constructed a linear prediction model (using the ridge regression algorithm^11^) to obtain the best correlation between the ILI rates gathered by the Royal College of General Practitioners (RCGP) and published by Public Health England (PHE)^a^ and the number of times each term was mentioned in tweets during the same time period. We then selected the 20 phrases which had the largest weight in the model, and retained only those tweets which contained one or more of these terms. The terms are listed in Table1. The correlation between the ILI rate and the predicted ILI rate was computed using 10-fold cross-validation^12^: the data were separated into 10 random partitions of the same size. A model was built using 9 of the partitions, and the correlation was measured for samples in the 10th partition. This was repeated 10 times, so that the correlation was measured once for each of the partitions. This procedure was used so as to reduce the bias of the estimated correlation. The average correlation (over the 10-fold) between the ILI rate and the predicted ILI rate using these 20 terms was 0·685.

**Table 1:** Terms used for the first stage of tweet filtering

Bad cough	Bed flu	Chest infection
Chesty cough	Cold flu	Cough
Cough syrup	Coughing	Feel sick
Flu	Food feel sick	Headache night
Illness	Man flu	Shivering
Throat cough	Vomit	Vomiting
Waking headache	Worst cough	

The resulting tweets could describe a specific person who is suffering from ILI or could be related to more general aspects of the flu, such as general observations on the fact that some people are ill, or calls to vaccinate. Therefore, we employed a second filter, which was aimed at identifying tweets that stated that the person posting them or one of their family members was suffering from ILI.

We constructed a training set for this classifier by randomly selecting 1500 tweets after the first stage of filtering. These tweets were labeled by five human assessors as to whether they refer to the poster of the tweet, their immediate family members, or none of the former. The assessors were recruited from the crowdsourcing Web site CrowdFlower.

Tweets were represented through four families of attributes:

1. A vector-space model with words, word pairs, and lexical affinities: Each phrase (word, word pair or lexical affinity) was represented as a single attribute, and the value of that attribute was the number of times that the phrase appeared in the tweet. Lexical affinities^13^ are word pairs within the same sentence separated by no more than five words.
2. Whether tweets contained one of six “emoticons,” that is, sequences of characters used to represent a smiling or a sad face, etc.
3. Whether tweets were a reply to another tweet.
4. Whether tweets contained a hashtag.

A linear support vector machine (SVM)^12^ classifier with probabilistic outputs was then trained to these data (libSVM^14^ with default settings). The SVM algorithm finds the best weights to give to each attribute so that a weighted sum over the attributes will give the probability that a tweet is referring to the poster of the tweet or their immediate family members. The classifier was applied to all tweets after the first stage of the filtering. The resulting probability estimates that a tweet contains a statement on the poster of the tweet or their family members were used as a weight in subsequent processing (see below). The performance of the classifier is analyzed in the Results.

### Identifying family-related tweets

In order to determine the secondary attack rate, we need to determine the probability that a user will tweet about a family member (see Section “Changes in fSAR over theinfluenza season” for details). To do so, we need to identify tweets that refer to family members.

Some Twitter users regularly report on a variety of aspects of life. Others use their account for specific purposes, for example, work-related topics. To distinguish between users who mention their immediate family members in their tweets from those who did not, we computed how frequently (if at all) family members were mentioned in each user's tweets. Tweets were identified as referring to family members if they contained one or more of the following keywords: “wife,” “husband,” “partner,” “hubby,” “girlfriend,” “boyfriend,” “son,” “daughter,” or “child.”

The demographic profile of Twitter users indicates fewer than 10% of users are under 18 years of age.^b^ Therefore, we did not include words related to parents in the list of family-related keywords.

In a random sample of 200 tweets, 67% of the tweets which contained one or more of these keywords referred to people's own family members.

### Estimating the familial secondary attack rate

We make the following basic assumptions: First, a Twitter user who has ILI may tweet about it. If she does, and if one of her immediate families also develops ILI, there is some probability that she will tweet about this fact as well. This assumption is supported by previous work,^15^,^16^ which found that it is possible to estimate influenza load from Twitter messages about influenza.

Formally, we denote the familial SAR by *P*_SAR_, and the probability that a user will report on a family member via her Twitter feed by *P*(*R*). Given that a Twitter user has reported suffering from ILI, the probability that he will report on a family member suffering from ILI is given by: 

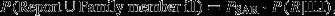
 where *P*(*R*|*ILI*) is the probability of reporting on the health of a family member conditioned on them suffering from ILI.

Our second assumption is that *P*(*R*|ILI) ≈ *P*(*R*); that is, the probability of reporting about ILI in a family member is approximately the same as reporting on other aspects related to the family member. We estimate *P*(*R*) for each user as the ratio of their tweets mentioning a family member to the number of all tweets they made, regardless of influenza.

A third assumption is that when both a Twitter user and their family member have experienced ILI within a few days, it is significantly more likely that one of them has infected the other, than the likelihood that they were both infected independently. Cases of the latter type may skew our estimate of fSAR.

Finally, we assume that if an ILI has been passed within a household, it can take no more than several days to develop. Here, we use a maximum of 7 days, as in Carcione et al. 5. If a Twitter user and a family member are reported suffering from ILI, but more than 7 days have passed between the two reports, we will assume that the source for contagion was different for the two individuals.

Under these assumptions, we can compute *P*_SAR_ from a population of users who reported suffering from ILI. We denote whether user *i* from the population reporting ILI also reported a family member suffering from ILI by *T*_*i*_, where 

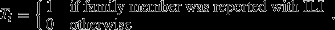


From the above, we note that *T*_*i*_∽*Bernoulli(P*_SAR_ · *P*_*i*_(*R*)) for each user. Given the population of users, the maximum likelihood value of *P*_SAR_ can be estimated using linear regression in the following manner^c^: Let *T*_*i*_ and *P*_*i*_(*R*) be the corresponding values for the *i*th user, where *i *= 1, 2, … *N*. We construct two column vectors *T* = [*T*_*i*_]_*i* = 1…*N*_ and P_R_ = [*P*_*i*_(*R*)]_*i* = 1…*N*_. Using the Moore-Penrose pseudo-inverse, the maximum likelihood estimation of *P*_SAR_ is given by: 

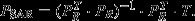


In this work, we employ weighted linear regression, weighting the data from each user according to the probabilities found by the above-mentioned classifier. In cases where users made several tweets referring to ILI symptoms in themselves, we compute the maximum of the probabilities assigned to these tweets. Similarly, we compute the maximum of the probabilities in ILI tweets referring to immediate family members. We used the maximum because a single, highly explicit, tweet mentioning specific ILI could indicate the fact that a person was ill (other methods for determining the user weightings are also possible, but were not investigated further). Then, we compute a confidence score in the tweets of each user by averaging these two probabilities (or take one, in cases where a user referred only to themselves or their family members). We denote this confidence by *w*_*i*_ for the *i*th user and use it to weight the data of each user. In a similar notation to the above, we construct *W*, a diagonal matrix of size *N*×*N*, where the *i*th element of the diagonal is equal to *w*_*i*_, then: 

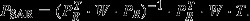


### Estimating the familial serial interval

We measured the interval between the report of when a Twitter user complained of ILI and when they reported that their family member had ILI, for those users who reported on both them and their family member suffering from ILI.

The familial serial interval (fSI) is the average of these intervals.

## Results

### Performance of the classifier used to identify specific ILI statements

The agreement between the labelers of the 1500 tweets labeled using crowdsourcing was such that in 97·5% of tweets, a majority of labelers agreed on the label. In 79·5%, there was an agreement of 4 or more of the labelers.

**Figure 1:**
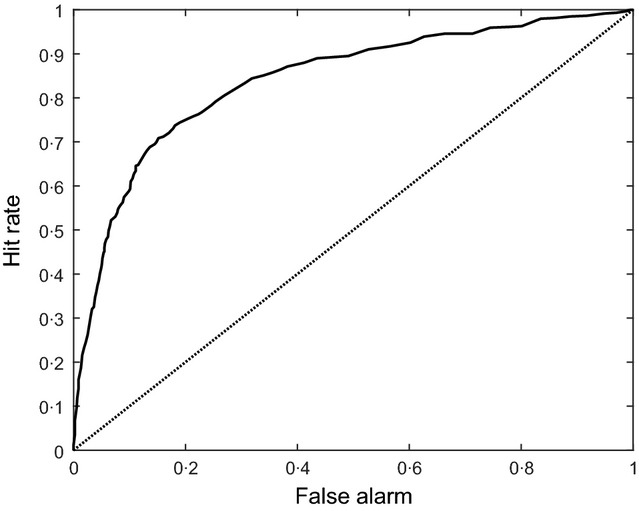
Receiver operating curve (ROC) of the classifier to distinguish personal from general tweets. The area under the ROC is 0·84.

We estimated the performance of the classifier using 50-fold cross-validation. The resulting receiver operating curve (ROC) is shown in Figure1. The area under the ROC is 0·84, implying that classification of the tweets can be considered relatively accurate. As noted above, we use the classifier to weight the examples in the estimation of the fSAR.

### User survey

As noted above, we conducted a survey among Twitter users and requested they report on whether they tweet about family members, and, separately, whether, if one of their family members is suspected of having the flu, would they tweet about it. Among users who tweeted about family matters, 83% reported they would tweet about a family member having the flu.

This suggests our assumption that *P*(*R*|*ILI*) ≈ *P*(*R*) is likely correct, as the vast majority of users who tweet about their family report their likelihood to tweet about a family member with ILI.

### Seasonal fSAR

Table2 shows the number of users who mentioned ILI symptoms. This table also shows the fSAR estimated for both seasons. The fSAR for the 2013 season is approximately 23% lower than that of the 2012 season. The 2013 season is known to have been less severe than the 2012 season: Doctor visits for ILI symptoms in England during the 2013 season were lower than the 2012 season, peaking at 8·7 per 100 000, compared to 32·7 for the 2012 season.^17^

**Table 2:** Familial secondary attack rate (fSAR) and data volumes for the 2012 and 2013 seasons

Season	Number of users reporting influenza-like illnesses symptoms	Familial secondary attack rate
2012	65 422	30·5% (SE 1·3%)
2013	93 459	25·7% (SE 0·8%)

Standard errors of fSAR estimate were computed using bootstrap sampling with replacement.

### Changes in fSAR over the influenza season

**Figure 2:**
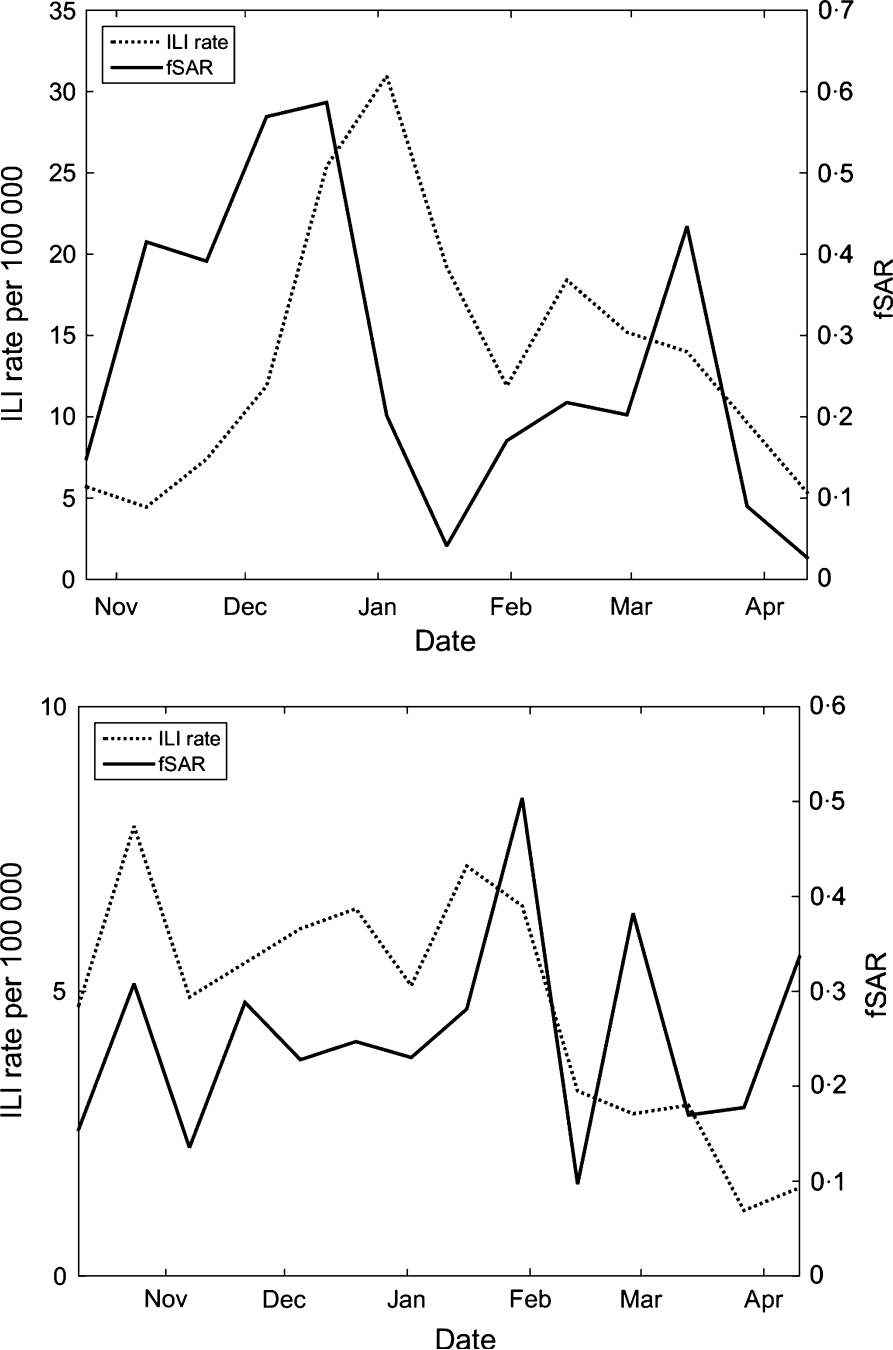
Influenza-like illnesses (ILI) rates (per 100 000, dotted, left axis) compared to the familial secondary attack rate probabilities (shown as a full line, right axis) over the influenza season for the 2012 (top) and 2013 (bottom) seasons.

In the previous section, we reported the fSAR computed from tweets collected over the entire influenza season. Here, we report the fSAR computed from tweets reported during 2-week intervals over the influenza season. We compare these to ILI rates reported by PHE^d^ in each of these intervals. The graphs for the two seasons are shown in Figure2. In the 2012 season, fSAR clearly begins to increase in advance to ILI rates do and appears simultaneous in the 2013 season, suggesting that higher transmissibility causes an uptick in ILI rates.

**Figure 3:**
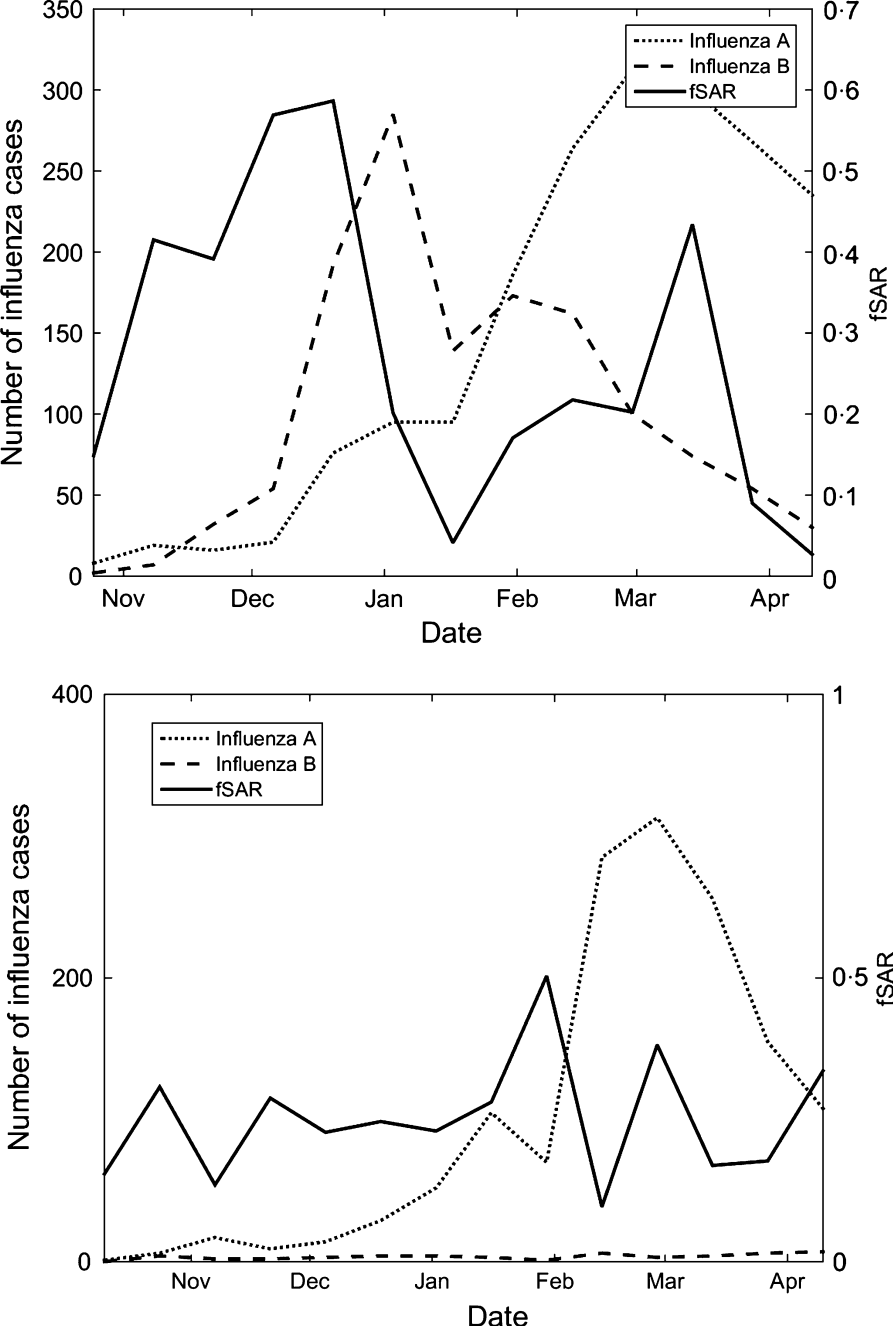
Estimated secondary attack rate compared to the number of confirmed cases influenza A (all strains) and B, from Public Health England data. The 2012–2013 season is shown on the top, and the 2013–2014 season on the bottom.

Figure3 compares estimated fSAR over time to virologically confirmed rates of influenza, as reported in the PHE DataMart system,^e^ a laboratory-based respiratory virus surveillance system.^18^ The 2012 season was unusual in terms of having an early wave of influenza B activity peaking at the beginning of January 2013, and a later wave of influenza A activity (mainly A(H3N2) but also some A(H1N1pdm2009)) peaking at the end of February. As the Figure shows, in the 2012 season, we observe a peak in fSAR closely timed with a peak of influenza B in January, and a later peak in fSAR overlapping a peak of influenza A activity in February. Peaks in fSAR seem to be correlated with the initial rise in influenza activities of each strain. Thus, the wave that peaks in December is likely driven by the wave of influenza B activity, and second peak is likely related to the influenza A wave. In the 2013 season, there were low levels of ILI. A(H1N1pdm2009) was the dominant strain which increased from the end of 2013 to peak at the end of February 2014. This likely relates to the peak of fSAR in January and February 2014. Once more, this indicates that influenza A had lower fSAR than that of influenza B.

These effects can be quantified using a regression model where the independent variables are the changes (derivatives) of the virological profiles and the dependent variable is fSAR. We have empirically determined that a stronger correlation appears for a quadratic form of fSAR, and these results are reported here.

Table3 shows the model parameters for each of the two seasons. First, we note the high *R*^2^ values of both models, which show that the models explain 34% and 45% of the variance. Second, the dominant strain (influenza B in 2012 and influenza A in 2013) in each season is statistically significantly correlated with fSAR. While only two seasons are represented here, this suggests increased fSAR is correlated with the beginning of a wave of influenza.

**Table 3:** Regression model between the slope of the virological profiles and a quadratic familial secondary attack rate

	Season	
	2012	2013
Influenza A coefficient	−0·0008	0·0005^*^
Influenza B coefficient	0·0013^*^	0·0071
Adjusted model *R*^2^	0·45^*^	0·34^*^

Stars denote statistically significant results (*P* < 0·05).

### Familial serial interval

Familial SI, the average difference in time between reports of ILI symptoms of a Twitter user and their family members (or vice versa), was 2·41 days during the 2012 season, and 2·48 during the 2013 season (not statistically significant, rank sum test). Thus, although fSAR differed significantly between seasons, the time to infection did not.

**Figure 4:**
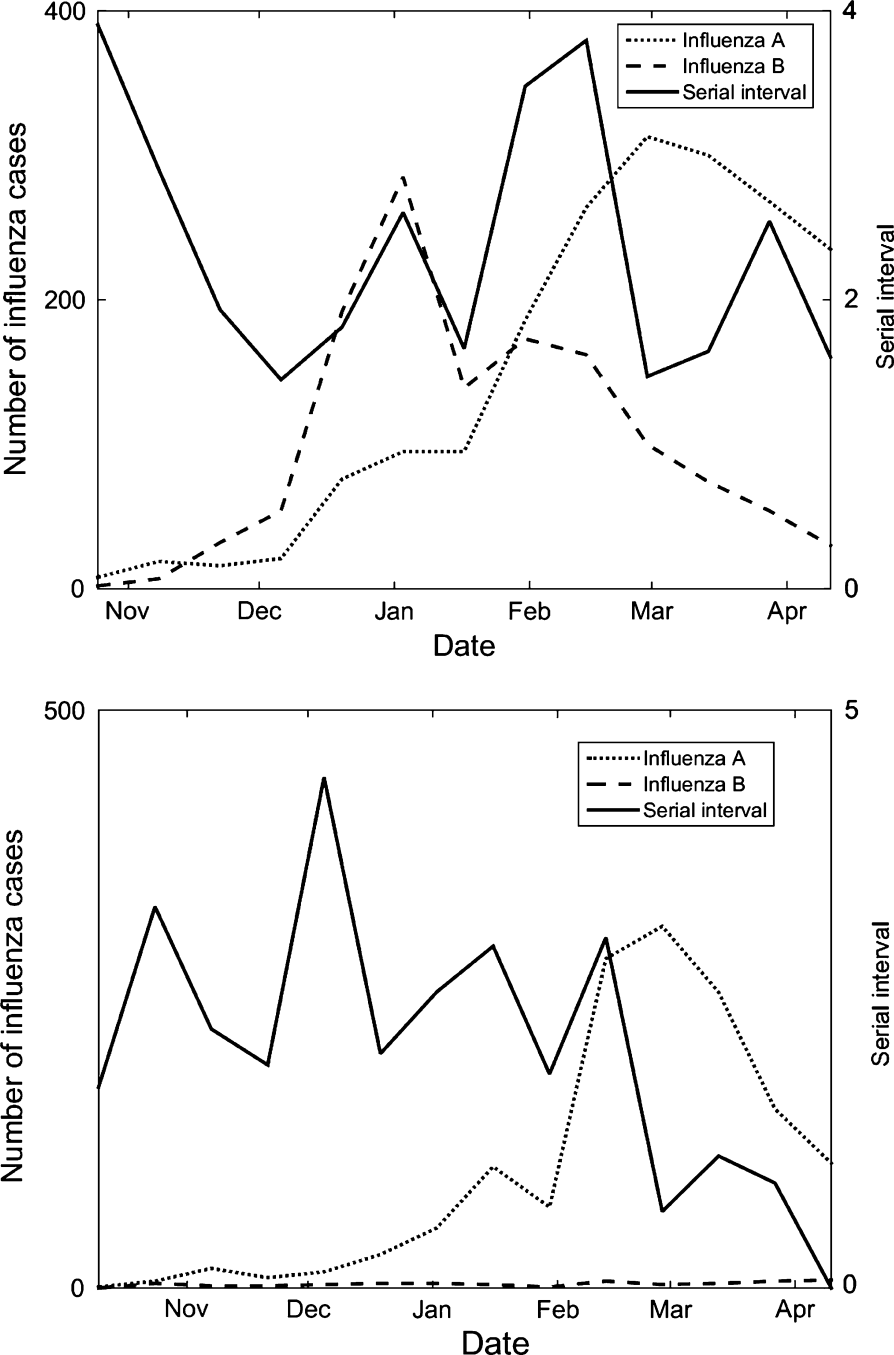
Estimated serial interval compared to the number of confirmed cases influenza A (all strains) and B, from Public Health England data. The 2012–2013 season is shown on the top, and the 2013–2014 season on the bottom.

Figure4 compares estimated fSI over time to the number of virologically confirmed cases of influenza in 2-week intervals. In the 2012 season, fSI seems to increase around the peak of the influenza B activity and increase before the peak of influenza A activity. However, models similar to those shown in Table3 do not reach statistical significance, indicating that additional factors beyond the virological profiles may be influencing fSI. Further research is required to understand these interactions and their effect on fSI.

## Discussion

The familial secondary attack rate and the SI are important parameters in understanding pandemic and seasonal influenza. However, collecting data for these estimates can be difficult, because of the need for continuous, large-scale tracking of susceptible populations or prospective identification and follow-up of cases and their contacts. Here, we demonstrated the ability to compute fSAR and fSI from social media.

Our work provides a repeatable method for assessing SAR and SI of different strains of ILI including seasonal and pandemic strains. This may be particularly important in assessing the potential threat of new strains, which could be identified through a sudden rise in fSAR or fSI. As we have shown (Figures2 and 3), such a rise appeared when a less-familiar strain (Influenza B) appeared in the 2012 season. Understanding these parameters can help to inform how health authorities need to intervene and is a component to assessing severity of the threat of a new strain and changes thereof.

Several previous studies attempted to estimate SAR and fSAR. Recent examples of pandemic influenza include an fSAR estimate for H1N1 of 7·6%^19^ and 11·3%.^20^ fSAR for the H5N1 strain was estimated at 29%.^21^ In the 2009 epidemic of H1N1, Carcione *et al*.^5^ estimated the fSAR of H1N1 in Western Australia at 27·9%. For seasonal influenza, Carcione *et al*.^5^ report fSAR is in the range of 10–40%, varying by demography, location and season.^5^ We note that SAR has different definitions, where a common one is the number of secondary cases divided by the number of people other than the index case (the first infected person in a family). Here, we report the proportion of households that have at least one secondary case. The difficulty in estimating the number of people residing in a household necessitates the use of fSAR as detailed in the Methods, but may cause the SAR estimation reported here to be higher than that computed using other definitions. We do not know of a published estimate for fSAR in England during the two seasons reported in this study. However, given past estimates, and the fact that the average family size in the UK is 2·4,^f^ our results of fSAR are within the established range for this parameter. Other evidence supports our estimate. First, the estimated fSAR for the 2013 season was substantially lower than that of the 2012 season, which fits with clinical findings for these seasons.

Second, as our figures show, the fSAR associated with the influenza B wave of the 2012 season was much higher than the peak fSAR relating to influenza A in that year. Variations in SAR may be driven by intrinsic diversity in viral transmissibility, as well as variations in population susceptibility. For example, in strains that have been in common circulation in recent years, including both influenza A(H1N1pdm2009) and influenza A(H3N2), high levels of immunity and low corresponding SAR could be expected. Conversely, population immunity to influenza B, which rarely causes major outbreaks, may be low and SAR correspondingly high.

Our findings of distinct peaks of SAR suggest that SAR is not a constant factor but is a dynamic phenomenon that may be driven by a combination of factors. For example, a downward swing in SAR may reflect the accumulation of immunity within the population through the course of an outbreak. One of the major advantages of the proposed method is the ability to rapidly estimate fSAR with a very low effort and negligible cost. The dynamic nature of fSAR necessitates this rapid estimation capability.

Our estimates of influenza SI (circa 2·5 days) are consistent with those identified from prospective studies of contacts of influenza.^22^ Here too, an additional advantage of our methods is the ability to provide information on the change in fSI throughout the season.

One of the causes for the overestimation of influenza rates by Google Flu trends, an Internet-based surveillance system, was media interest in influenza, which caused a people to search more often for influenza-related information, skewing its estimate for the number of people suffering from ILI.^9^ We posit that the methods proposed here for the estimation of fSAR and fSI are more immune to these errors, because a heightened awareness and interest in influenza will similarly affect the likelihood of reporting on both index and secondary cases, thus leaving the estimates of fSAR and fSI unaffected.

Future work will utilize other sources of Internet data, including search engine queries and other social media, to validate and improve the estimate for fSAR. Additionally, targeted advertisements could be used to invite people reporting ILI symptoms to provide samples for virological testing, thus providing ground-truth data with minimal delay.

### Limitations

One limitation of our method pertains to the demographic profile of Twitter users. Such users are not, currently, representative of the population. For example, as noted in the Methods section, fewer than 10% of users are under 18 years of age. As fSAR is known to vary with demography, if a specific influenza strain affects, for example, children, such strains may be underrepresented in the data and hence bias our estimation. However, the variations in fSAR as a function of demography are relatively small (e.g., between 12·5 and 16·3, as a function of household size^5^), these differences should not, in general, cause a significant bias in the estimate of fSAR.

Another potential bias of our method stems from the fact that we do not have an explicit identification of the index case versus those of the household contacts. Thus, if two members of a household were independently infected (as can happen when the infection rate is high), this may count as a secondary attack, thus skewing our results. Previous researchers implicitly distinguished index cases from household cases according to the timing of infection, for example, by not considering cases with the same symptom onset.^5^ However, anecdotal evidence suggests that some people refer to ILI in both them and their family members in the same tweet.

## Footnotes

a https://www.gov.uk/government/publications/annual-flu-reports

b http://royal.pingdom.com/2012/08/21/report-social-network-demographics-in-2012/

c An alternative estimation is to maximize the log-likelihood of the product of two Bernoulli variables. Details are provided in the Appendix A. Here, we use the linear estimator which gives similar values, but allows the natural addition of a weighting function, as detailed below.

d https://www.gov.uk/government/statistics/weekly-national-flu-reports

e https://www.gov.uk/government/statistics/weekly-national-flu-reports

f See: http://www.ons.gov.uk/ons/rel/family-demography/families-and-households/2013/stb-families.html If each household with a secondary case contains one index case, the ratio of fSAR computed according to our estimate compared to that computed in Carcione et al., 2011^5^ is 1/1.4.

## Appendix A Estimation of SAR using a solution to the Bernoulli log-likelihood function

As noted in Section “Changes in fSAR over the influenza season”, *T*_*i*_ ∽ *Bernoulli(P*_SAR_ · *P*_*i*_(*R*)) for each user. In that section, we estimated using linear regression. An alternative is to maximize the log-likelihood function of a Bernoulli distribution, as follows: Let *L*(*P*) be the likelihood function for a product of two Bernoulli variables, *P*_SAR_ and *Pi*(*R*).

The log-likelihood of *L*(*P*) can be maximized numerically for a given set of data points.

Although the error criterion for linear regression and this function differ, the results in practice are similar. Hence, we opt for the use of linear regression, where weights of different data points can be included naturally.
